# Controlling Epileptic Activity by Modulating Putrescine Metabolism

**DOI:** 10.3390/biom16091329

**Published:** 2026-09-12

**Authors:** Saif Qahtan, Zsolt Kovács, Enikő Rauch, Pál Szabó, László Héja

**Affiliations:** 1 Institute of Organic Chemistry, HUN-REN Research Centre for Natural Sciences, Magyar Tudósok Körútja 2, 1117 Budapest, Hungary; saif.qahtan@qu.edu.iq; 2 Hevesy György PhD School of Chemistry, ELTE Eötvös Loránd University, 1117 Budapest, Hungary; 3 College of Science, University of Al-Qadisiyah, Al-Diwaniyah 58001, Iraq; 4 Department of Biology, ELTE Eötvös Loránd University, BDTTC, Károlyi Gáspár tér 4, 9700 Szombathely, Hungary; kovacs.zsolt@sek.elte.hu (Z.K.);; 5 Institute of Biology, University of Pécs, Ifjúság 6, 7624 Pécs, Hungary; 6 Centre for Structural Science, HUN-REN Research Centre for Natural Sciences, Magyar Tudósok Körútja 2, 1117 Budapest, Hungary; szabo.pal@ttk.hu

**Keywords:** epilepsy, putrescine, Glu-GABA exchange, spermidine synthase, monoamine oxidase

## Abstract

Glial mechanisms regulate neuronal excitability through multiple mechanisms, including the control of extracellular inhibitory signaling. One such mechanism is the Glu/GABA exchange process, in which glutamate uptake is coupled to GABA release, with GABA being synthesized from the polyamine putrescine, placing putrescine at a central position in metabolic pathways that may influence epileptiform activity. Here, we examined how pharmacological manipulation of key enzymes of putrescine metabolism affects seizure-like activity in the low-[Mg^2+^] in vitro model of frontotemporal epilepsy, complemented by in vivo recordings in the non-convulsive absence epilepsy model Wistar Albino Glaxo/Rijswijk (WAG/Rij) rats. Increasing putrescine availability by inhibiting spermidine synthase with trans-4-methylcyclohexylamine significantly reduced both the duration and appearance of seizure-like events. Strikingly, simultaneous inhibition of monoamine oxidase B (MAO-B) and diamine oxidase (DAO) with deprenyl and aminoguanidine almost completely abolished seizure-like events in vitro and markedly suppressed spike–wave discharges in WAG/Rij rats. This effect was largely reversed by blockade of GAT-2/3 transporters with SNAP-5114, suggesting the presence and significant anti-epileptic potential of a MAO-B- and DAO-independent putrescine–GABA synthesis pathway. Together, these findings indicate that the anticonvulsant effects of putrescine metabolism arise from the coordinated engagement of multiple parallel pathways rather than from a single dominant enzymatic route. Putrescine thus appears to function as a metabolic hub whose increased availability can be channeled into several anticonvulsant processes, suggesting that therapeutic strategies enhancing putrescine-dependent inhibitory pathways at the network level may offer promising avenues for the modulation of epileptiform activity.

## 1. Introduction

Epilepsy is characterized by recurrent episodes of abnormal neuronal synchronization that arise from an imbalance between excitatory and inhibitory signaling in the brain. Anti-epileptic drugs (AEDs) exert their therapeutic effects by modulating neuronal signaling pathways and targeting key elements of neuronal excitability and neurotransmission, resulting in seizure suppression and improved seizure control in patients with epilepsy [1,2]. Generally, AEDs work via numerous mechanisms, such as blocking voltage-gated sodium channels, inhibiting voltage-gated calcium channels, antagonizing glutamate receptors, or modulating voltage-gated potassium channels [1,3,4]. Among these mechanisms, enhancement of GABAergic inhibition remains one of the most effective strategies in limiting neuronal hyperexcitability [5,6,7]. While most anti-epileptic therapies target neuronal receptors or ion channels, increasing evidence indicates that astrocytes also play a critical role in regulating network excitability [8,9,10]. Astrocytes participate in neurotransmitter clearance, metabolic support, and ion homeostasis, and their dysfunction has been implicated in the initiation and maintenance of epileptic activity. In particular, astrocytes can actively influence inhibitory signaling through the regulation of extracellular GABA levels.

We have previously revealed that during epileptiform activity, a major source of extracellular GABA is the astrocytic Glu/GABA exchange mechanism, by which the uptake of Glu is directly converted to GABA release via the concerted action of astrocytic Glu and GABA transporters [10,11]. The released GABA has been shown to be synthesized from putrescine (PUT) [11,12]. Recently, we have also demonstrated that direct application of PUT shortens epileptiform activity both in vitro and in vivo [13]. In addition, increasing astrocytic PUT concentration by blocking the PUT-metabolizing enzyme spermidine synthase was found to be effective against spike–wave discharges (SWDs) in Wistar Albino Glaxo/Rijswijk (WAG/Rij) rats, an established model of non-convulsive absence epilepsy [10]. These findings suggest that putrescine metabolism plays an important role in regulating inhibitory signaling during epileptic activity.

Putrescine occupies a central position in cellular polyamine metabolism. In astrocytes, it can either be converted to higher polyamines such as spermidine or serve as a substrate for GABA synthesis. Because these metabolic routes compete for the same substrate, changes in putrescine metabolism may strongly influence the availability of astrocytic GABA and thereby affect neuronal excitability. Despite the growing recognition of these pathways, it remains unclear which steps of putrescine metabolism can be effectively targeted to modulate epileptiform activity. In particular, it is not known whether seizure activity can be altered by manipulating specific enzymatic steps in putrescine synthesis or degradation, or whether the anticonvulsant effects of this metabolic system arise from broader changes in putrescine availability.

Here we investigated how pharmacological manipulation of key enzymes in putrescine metabolism influences epileptiform activity primarily in the low-[Mg^2+^] in vitro model of frontotemporal epilepsy. We first examined whether increasing putrescine availability by spermidine synthase inhibition is an effective strategy against convulsive epileptiform activity by measuring the anti-epileptic effects of trans-4-methylcyclohexylamine (4-MCHA), a specific spermidine synthase inhibitor, in hippocampal slices. We then investigated the consequences of inhibiting different putrescine metabolism pathways through monoamine oxidase B, diamine oxidase and ornithine decarboxylase. In addition, we used in vivo EEG recordings in WAG/Rij rats as a complementary model to assess how these pharmacological manipulations affect spike–wave discharges in a non-convulsive absence epilepsy paradigm. By mapping how these interventions affect seizure-like activity in vitro and spike–wave discharges in vivo, we aimed to determine whether specific steps of putrescine metabolism represent effective targets for modulating network excitability at the systems level.

## 2. Materials and Methods

### 2.1. Animals

Experiments were carried out according to the instructions of the Hungarian Act of Animal Care and Experimentation (1998, XXVIII), European Communities Council Directive (86/609/EEC) and EU Directive 2010/63/EU as well as the approval of the Animal Care and Experimentation Committee of the Eötvös Loránd University (BDTTC) and National Scientific Ethical Committee on Animal Experimentation (Hungary; license number: VA/ÉBÁF-ÁO/00279-4/2021). Male WAG/Rij rats (*n* = 16; 10 months old, 294–309 g) were housed in groups (4 animals in every group; BDTTC, Eötvös Loránd University), and 17 Wistar rats of both sexes (P9-15) were used for the in vitro measurements. All animals were kept under standard laboratory conditions (12:12 h light-dark cycle; light was on between 08.00 AM and 08.00 PM; free access to food/SSNIFF RM-Z + H rat breeding and maintenance diet, TOXI-COOP Ltd., Budapest, Hungary, and water; air-conditioned room at 22 ± 2 °C). The rats were euthanized by isoflurane after the last treatment. All efforts were made to minimize pain, suffering and the number of animals used.

### 2.2. Solutions

Artificial cerebrospinal fluid (ACSF) contained in mM: 129 NaCl; 3 KCl; 1.6 CaCl_2_; 1.8 MgSO_4_; 1.25 NaH_2_PO_4_; 21 NaHCO_3_; 10 glucose. To induce epilepsy, MgSO_4_ was omitted and 2 mM KCl was added (low-[Mg^2+^] ACSF). In the in vitro experiments, 4-MCHA (200 µM), deprenyl (10 µM), aminoguanidine (100 µM) and DFMO (2 mM) were diluted in normal or low-[Mg^2+^] ACSF. SNAP-5114 was diluted in ethanol in stock concentration of 100 mM and diluted to the final concentration in normal or low-[Mg^2+^] ACSF. The pH value of 7.4 was not affected by the applied concentrations. All solutions were continuously oxygenated (95% O_2_, 5% CO_2_).

For the in vivo measurements, aminoguanidine (150 mg/kg) and deprenyl (15 mg/kg) were dissolved in saline. SNAP-5114 (20 mg/kg) was diluted in 10% dimethyl sulfoxide (DMSO) solution. All compounds were injected i.p. into WAG/Rij rats.

### 2.3. In Vitro Slice Preparation

Rat brain slices for low-[Mg^2+^] epilepsy model measurements: hippocampal–entorhinal slices from 9–15-day-old Wistar rats (Toxicoop, Budapest, Hungary). Transverse, 400 μm thick slices were cut in modified ACSF (75 mM sucrose; 87 mM NaCl; 2.5 mM KCl; 0.5 mM CaCl_2_; 7 mM MgSO_4_; 1.25 mM NaH_2_PO_4_; 25 mM NaHCO_3_; 25 mM glucose) at 4 °C using a vibratome (VT1000S; Leica, Nussloch, Germany) and incubated for recovery in a normal ACSF solution as described previously, gassed with 5% CO_2_/95% O_2_, pH 7.4, at 35 °C for 1 h, followed by another hour of incubation at room temperature. Slices were placed in a recording flow chamber and superfused continuously with oxygenated ACSF at room temperature (23–24 °C, 1 mL/min).

### 2.4. In Vitro Electrophysiology

Field potential recordings were performed at 31 °C using glass microelectrodes (1 to 4 MΩ), filled with ACSF solution, and inserted in the CA3 stratum pyramidale. Signals were recorded with Multiclamp 700A amplifiers (Axon Instruments, Foster City, CA, USA), low-pass filtered at 2 kHz and digitized at 10 kHz (Digidata 1320A, Axon Instruments). Recordings were analyzed after high-pass filtering at 1 Hz. Epileptiform activity was induced by switching the perfusing solution (ACSF) to low-[Mg^2+^] ACSF.

### 2.5. EEG Electrode Implantation, Recording and Evaluation

Stainless steel screw electrodes were chronically implanted epidurally under isoflurane–air mixture (2.0–2.5%) anesthesia into WAG/Rij rats. Electrodes were placed above the frontal cortex and parietal cortex based on stereotaxic coordinates (AP 2.0 mm and L 2.1 mm as well as AP −6.5 mm and L 2.1 mm, respectively) [14]. Moreover, reference and ground electrodes were placed above the cerebellar cortex. Dentacrylate cement (Ivoclar, Schaan, Liechtenstein) was applied to fix electrodes to the skull bones and Lidocaine ointment (5%; EGIS, Budapest, Hungary) was used to mitigate post-operative pain.

After a 2-week recovery period, EEG recording was carried out by a differential biological amplifier (Bioamp4, Supertech Ltd., Pécs, Hungary) and a data capture and analysis device (CED 1401 mkII, Cambridge Electronic Design Ltd., Cambridge, UK) between 1.30 PM and 5.00 PM. The sampling rate was 500 Hz, whereas the bandwidth of the EEG recording was 0.3 Hz to 150 Hz. Handling-evoked stress may induce changes in SWD number [15]. Thus, similarly to our previous studies [10,13] SWD number, SWD duration, time spent in SWDs and interictal interval length were evaluated between 30 and 210 min of recording periods. Moreover, 30 min sections of EEG recordings were evaluated separately by automated separation of SWDs [10] (all automatically selected SWDs were confirmed by manual supervision). SWDs can be characterized by 1–50 s duration, 7–11 Hz discharge frequency within SWDs and 0.2–1.0 mV amplitude and a train of asymmetric spikes and slow waves starting and ending with sharp spikes [15]. Moreover, the average amplitude of SWDs is at least twice as high as the basal EEG activity.

### 2.6. Data Processing

The sample sizes were based on our previous work in similar models [10,13] and on typical effect sizes observed for pharmacological interventions in the low-[Mg^2+^] and WAG/Rij models. Our goal was to achieve sufficient power to detect changes of approximately 30–40% in SLE or SWD measures with α = 0.05, which in practice led to *n* values in the range of 6–10 slices or 4–8 animals per group, consistent with the existing literature.

In vitro recordings were analyzed using pClamp 10.4 software (Axon Instruments) after high-pass filtering at 1 or 2 Hz. As it was not fully developed, the first SLE in each slice (SLE0) was discarded from data evaluation.

No blinding was applied during in vitro and in vivo experimental acquisitions due to the nature of the workflow. However, blinding was implemented at the data analysis stage by coding recorded files and treatment conditions prior to analysis, ensuring that signal processing and quantification were performed without knowledge of group identity.

All statistical analyses were performed using MATLAB version 9.13.0 (R2022b), OriginPro 10.1 (version 2024), and GraphPad Prism (version 8.4.3 (471), San Diego, CA, USA). Data are reported as mean ± SEM.

## 3. Results

### 3.1. Inhibition of Putrescine Catabolism Suppresses Epileptiform Activity

We have previously shown that application of exogenous putrescine decreases the duration of seizures both in vivo in the WAG/Rij absence epilepsy model and in vitro in the low-[Mg^2+^] model of frontotemporal epilepsy [13] and that this anti-epileptic effect is mediated by the astrocytic Glu/GABA exchange mechanism. PUT in astrocytes can be metabolized to either GABA or spermidine. Since the anti-epileptic activity of putrescine requires putrescine to be converted to GABA, we investigated whether inhibiting PUT catabolism to spermidine can be a viable strategy to enhance the anticonvulsive potential of the Glu/GABA exchange by providing additional putrescine as a source for GABA synthesis. To this end, we applied 200 µM 4-MCHA, a specific inhibitor of spermidine synthase [16], which we previously demonstrated to be able to increase brain GABA concentration [10].

**Figure 1:**
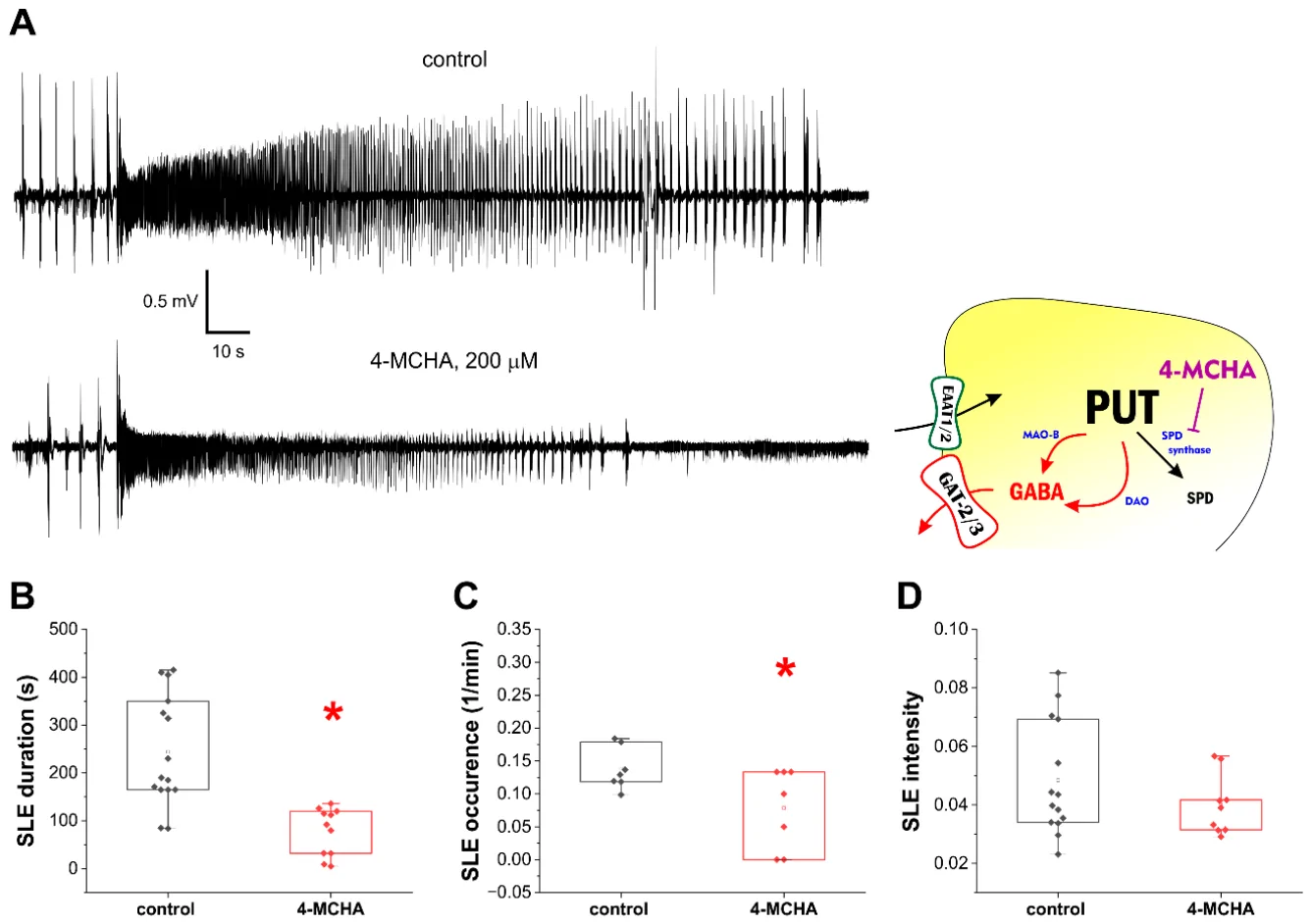
Inhibition of spermidine synthase suppresses epileptiform activity in the low-[Mg^2+^] epilepsy model. (**A**) Representative extracellular field potential recordings of seizure-like events (SLEs) induced by low-[Mg^2+^] ACSF in the CA3 region of a hippocampal slice under control condition (top) and the subsequent SLE observed after application of 200 µM 4-MCHA (bottom). (**B**) Average duration of SLEs under control conditions (black) and in the presence of 200 µM 4-MCHA (red). Each point represents the duration of a single SLE. (**C**) Number of SLEs per minute under control conditions (black) and in the presence of 200 µM 4-MCHA (red). Each point represents the SLE occurrence in a single slice. (**D**) SLE intensity, defined as the standard deviation of the field potential under control conditions (black) and in the presence of 200 µM 4-MCHA (red). Each point represents the SLE intensity in a single slice. Box edges represent 25th and 75th percentile, open squares represent means. Asterisk denotes significant difference between control conditions and 4-MCHA application.

We observed that the duration of seizure-like events (SLEs) in the low-[Mg^2+^] epilepsy model significantly decreased from 244 ± 30 s measured under control conditions to 78 ± 15 s in the presence of 200 µM 4-MCHA (*n* = 7 slices from 3 animals, *p* < 0.001) (Figure 1A,B). In addition, the number of SLEs also significantly decreased from 0.138 ± 0.012/min to 0.079 ± 0.023/min following 4-MCHA application (*n* = 7 slices from 3 animals, *p* = 0.04) (Figure 1C). Intensity of SLEs, defined as the standard deviation of the field potential recording, decreased only slightly, but not significantly, from 0.048 ± 0.005 measured under control conditions to 0.040 ± 0.003 in the presence of 200 µM 4-MCHA (*n* = 7 slices from 3 animals, *p* = 0.24) (Figure 1D).

### 3.2. Short-Term Inhibition of MAO-B Attenuates Epileptiform Activity

**Figure 2:**
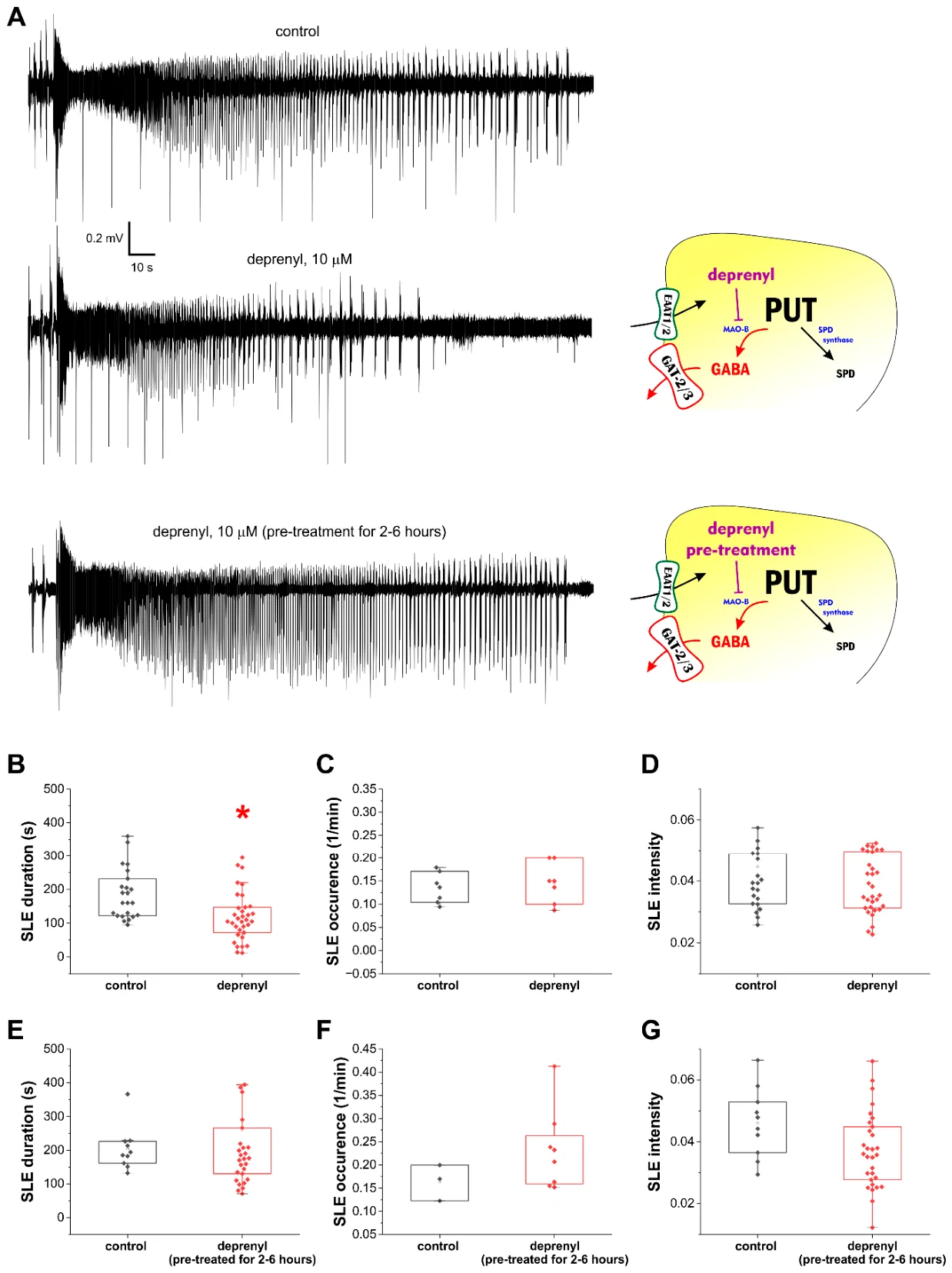
Short-term inhibition of monoamine oxidase B suppresses epileptiform activity in the low-[Mg^2+^] epilepsy model. (**A**) Representative extracellular field potential recordings of seizure-like events (SLEs) induced by low-[Mg^2+^] ACSF in the CA3 region of a hippocampal slice under control condition (top), after application of 10 µM deprenyl (middle) and after long-term pre-treatment of the slice with 10 µM deprenyl (bottom). (**B**) Average duration of SLEs under control conditions (black) and after application of 10 µM deprenyl (red). Each point represents the duration of a single SLE. (**C**) Number of SLEs per minute under control conditions (black) and after application of 10 µM deprenyl (red). Each point represents the SLE occurrence in a single slice. (**D**) SLE intensity, defined as the standard deviation of the field potential under control conditions (black) and after application of 10 µM deprenyl (red). Each point represents the SLE intensity in a single slice. (**E**) Average duration of SLEs in control slices (black) and in slices pre-treated with 10 µM deprenyl for 2–6 h (red). Each point represents the duration of a single SLE. (**F**) Number of SLEs per minute in control slices (black) and in slices pre-treated with 10 µM deprenyl for 2–6 h (red). Each point represents the SLE occurrence in a single slice. (**G**) SLE intensity, defined as the standard deviation of the field potential in control slices (black) and in slices pre-treated with 10 µM deprenyl for 2–6 h (red). Each point represents the SLE intensity in a single slice. Box edges represent 25th and 75th percentile, open squares represent means. Asterisk denotes significant difference between control conditions and deprenyl application.

After demonstrating that the anti-epileptic activity of the Glu/GABA exchange can be enhanced by increasing the putrescine–GABA synthetic pathway, we assessed whether epileptiform activity can be modulated by altering the amount of GABA produced from putrescine. First, we investigated the major pathway by which putrescine is converted to GABA by blocking monoamine oxidase B (MAO-B) with its potent, selective inhibitor deprenyl [17]. Application of 10 µM deprenyl significantly decreased the duration of SLEs from 186 ± 16 s measured under control conditions to 119 ± 12 s (*n* = 8 slices from 2 animals, *p* = 0.001) (Figure 2A,B). The number of SLEs and SLE intensity did not change significantly (Figure 2C,D) (*n* = 8 slices from 2 animals, *p* = 0.59 and *p* = 0.21, respectively).

Since we expected epileptiform activity to increase after applying a GABA synthesis inhibitor, we investigated whether this unexpected behavior was due to the inability of short-term deprenyl application to deplete the astrocytic GABA pool or whether deprenyl in this concentration may have MAO-B-independent actions, including effects on catecholamine reuptake and membrane excitability [18,19]. To this end, we measured the long-term effect of the inhibition of PUT-GABA conversion by pre-treating hippocampal slices with 10 µM deprenyl for 2 to 6 h to allow depletion of the GABA pool synthesized from PUT. Long-term pre-treatment of the slices with deprenyl did not produce any significant changes in the duration (Figure 2E), number (Figure 2F) or intensity (Figure 2G) of SLEs (*n* = 10 slices from 2 animals, *p* = 0.48, *p* = 0.24 and *p* = 0.056, respectively) compared to the control (*n* = 4 slices from 2 animals), suggesting that the short-term effect of deprenyl may correspond to the increased PUT level per se, not to its conversion to GABA. Although we cannot exclude that MAO-B-independent effects, rather than putrescine accumulation, contribute to the acute reduction in SLE duration, they would be expected to persist rather than disappear during long-term pre-treatment; therefore, we regard putrescine accumulation as the more likely explanation for the acute effect.

### 3.3. Blockade of DAO Does Not Affect Epileptiform Activity

**Figure 3:**
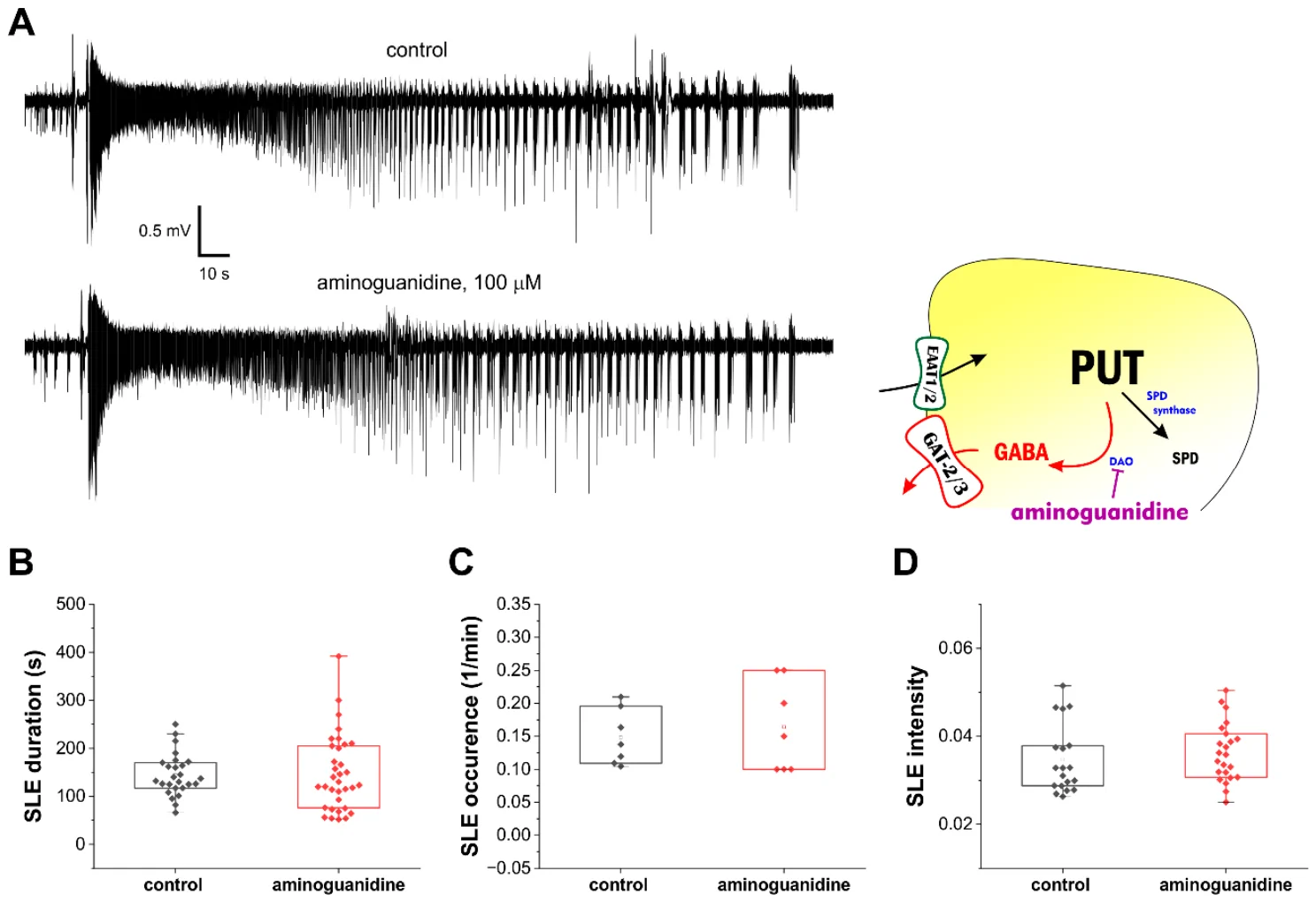
Specific inhibition of diamine oxidase does not affect epileptiform activity in the low-[Mg^2+^] epilepsy model. (**A**) Representative extracellular field potential recordings of seizure-like events (SLEs) induced by low-[Mg^2+^] ACSF in the CA3 region of a hippocampal slice under control condition (top) and after application of 100 µM aminoguanidine (bottom). (**B**) Average duration of SLEs under control conditions (black) and after application of 100 µM aminoguanidine (red). Each point represents the duration of a single SLE. (**C**) Number of SLEs per minute under control conditions (black) and after application of 100 µM aminoguanidine (red). Each point represents the SLE occurrence in a single slice. (**D**) SLE intensity, defined as the standard deviation of the field potential under control conditions (black) and after application of 100 µM aminoguanidine (red). Each point represents the SLE intensity in a single slice. Box edges represent 25th and 75th percentile, open squares represent means.

Although the major route of synthesizing GABA from putrescine in astrocytes is through MAO-B, an alternative pathway also exists in which diamine oxidase (DAO) directly oxidizes putrescine to γ-aminobutyraldehyde, followed by conversion to GABA by aldehyde dehydrogenase 1 family member A1 (ALDH1A1) [20]. To test whether this pathway may be responsible for the synthesis of GABA that is released by the Glu/GABA exchange mechanism, we measured SLEs in the low-[Mg^2+^] epilepsy model in the presence of aminoguanidine, a specific DAO inhibitor [21]. DAO blockade by 100 µM aminoguanidine did not produce any significant change in the duration (Figure 3A,B), number (Figure 3C) or intensity (Figure 3D) of SLEs (*n* = 7 slices from 2 animals, *p* = 0.45, *p* = 0.62 and *p* = 0.48, respectively).

**Figure 4:**
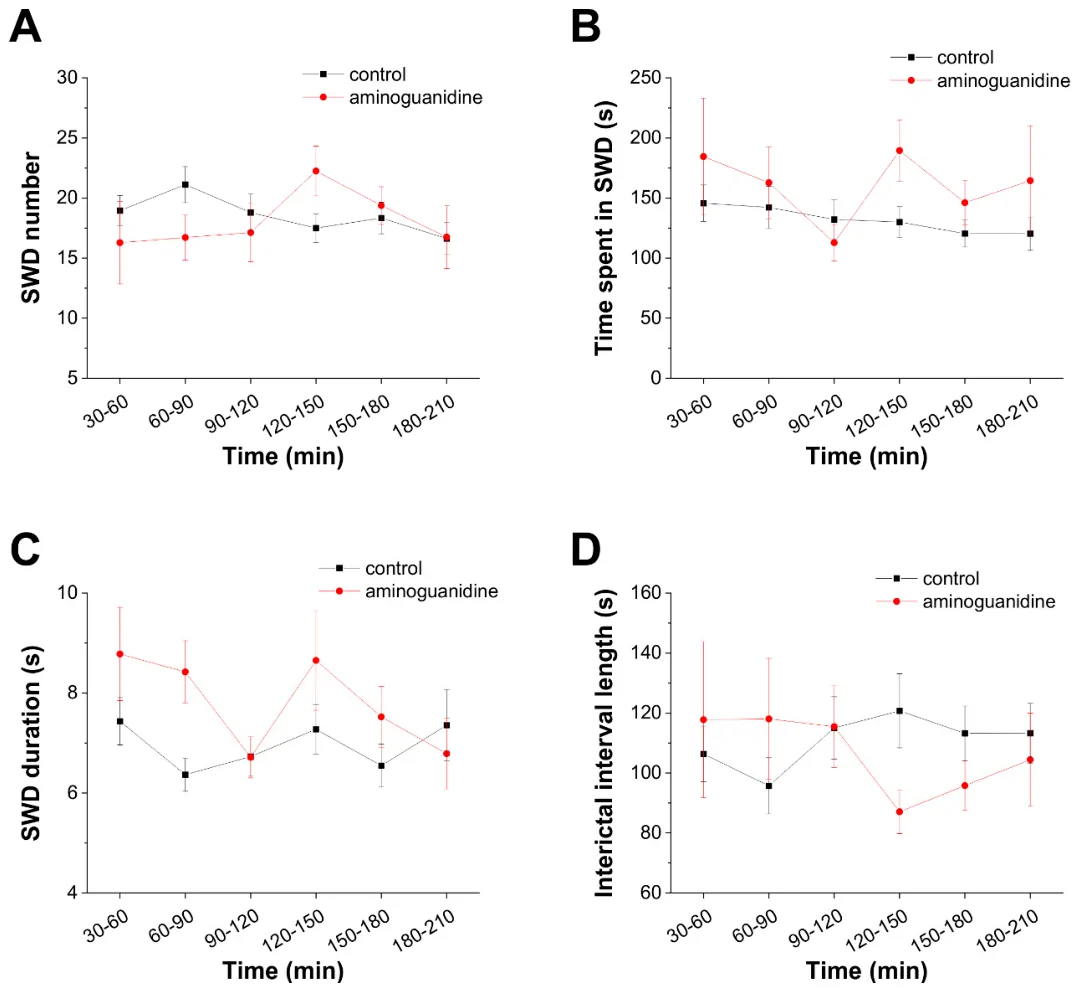
Specific inhibition of diamine oxidase does not affect epileptiform activity in the in vivo WAG/Rij rat model of human absence epilepsy. Effect of aminoguanidine (150 mg/kg, i.p.) on SWD number (**A**), total time spent in SWDs (**B**), SWD duration (**C**) and interictal interval length (**D**).

To assess the effect of long-term depletion of DAO-dependent astrocytic GABA pool on epileptic activity, we measured SWDs in vivo in WAG/Rij rats after 0.5–3 h of intraperitoneal (i.p.) treatment of the animals (*n* = 8) with 150 mg/kg aminoguanidine. Aminoguanidine did not change any of the investigated SWD parameters, such as SWD number, total time spent in SWD, SWD duration and interictal interval length (Figure 4).

### 3.4. Simultaneous Inhibition of MAO-B and DAO Pathways Eliminates Epileptiform Activity

MAO-B and DAO pathways can mutually compensate for each other [22]; therefore, the effect of inhibiting one pathway can be masked by the activation of the other route. To assess the genuine role of putrescine–GABA conversion in seizure control, we investigated whether blockade of GABA synthesis from putrescine affects epileptiform activity. To this end, we simultaneously inhibited MAO-B and DAO by their specific inhibitors, deprenyl and aminoguanidine, respectively, in both the low-[Mg^2+^] epilepsy model and in WAG/Rij rats.

**Figure 5:**
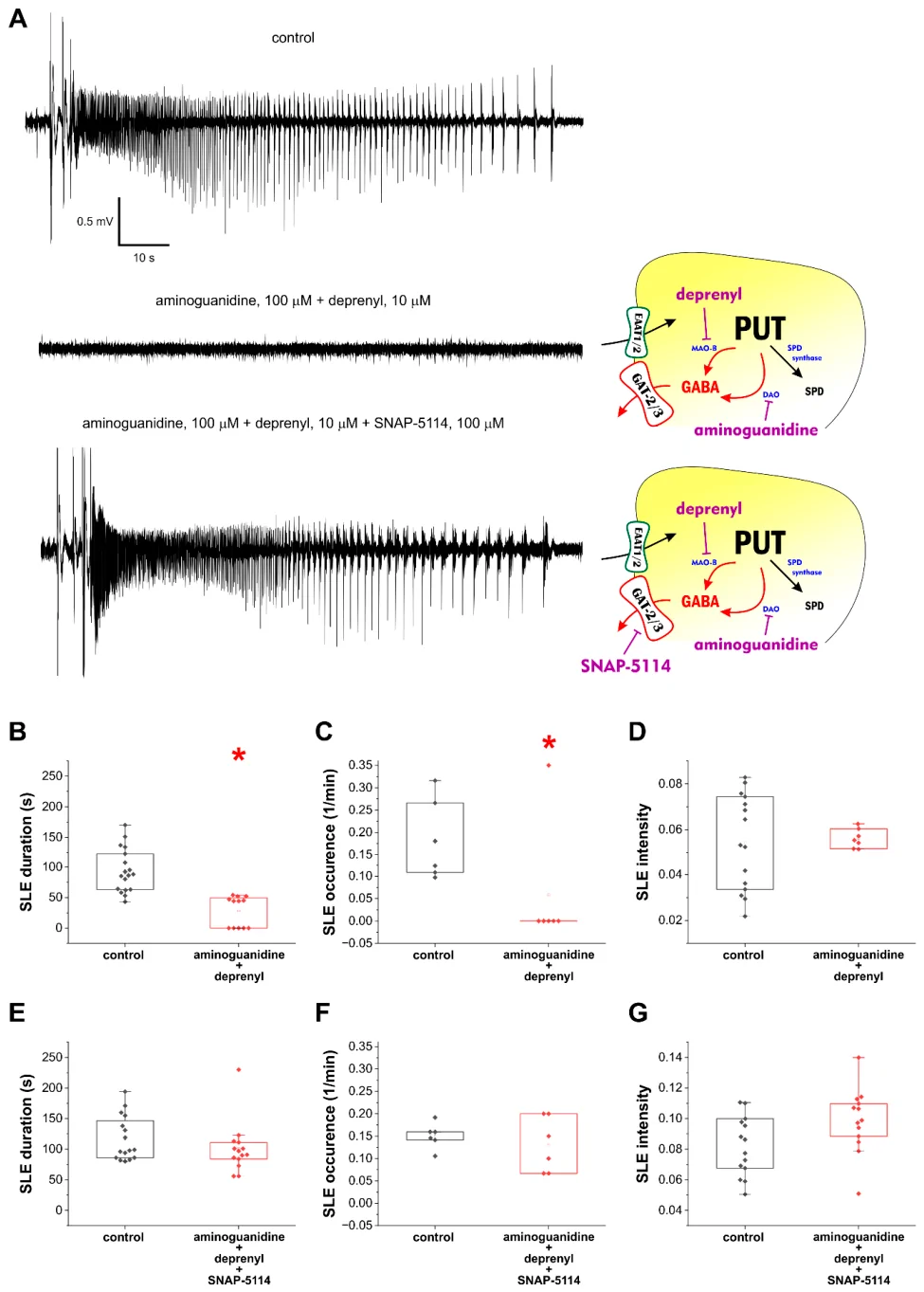
Simultaneous inhibition of monoamine oxidase B and diamine oxidase eliminates epileptiform activity and suggests the presence of an additional pathway for putrescine–GABA conversion in the low-[Mg^2+^] epilepsy model. (**A**) Representative extracellular field potential recordings of seizure-like events (SLEs) induced by low-[Mg^2+^] ACSF in the CA3 region of a hippocampal slice under control condition (top), after application of 100 µM aminoguanidine and 10 µM deprenyl (middle) and after application of 100 µM aminoguanidine, 10 µM deprenyl and 100 µM SNAP-5114 (bottom). (**B**) Average duration of SLEs under control conditions (black) and in the presence of 100 µM aminoguanidine and 10 µM deprenyl (red). Each point represents the duration of a single SLE. (**C**) Number of SLEs per minute under control conditions (black) and in the presence of 100 µM aminoguanidine and 10 µM deprenyl (red). Each point represents the SLE occurrence in a single slice. (**D**) SLE intensity, defined as the standard deviation of the field potential under control conditions (black) and in the presence of 100 µM aminoguanidine and 10 µM deprenyl (red). Each point represents the SLE intensity in a single slice. (**E**) Average duration of SLEs under control conditions (black) and in the presence of 100 µM aminoguanidine, 10 µM deprenyl and 100 µM SNAP-5114 (red). Each point represents the duration of a single SLE. (**F**) Number of SLEs per minute under control conditions (black) and in the presence of 100 µM aminoguanidine, 10 µM deprenyl and 100 µM SNAP-5114 (red). Each point represents the SLE occurrence in a single slice. (**G**) SLE intensity, defined as the standard deviation of the field potential under control conditions (black) and in the presence of 100 µM aminoguanidine, 10 µM deprenyl and 100 µM SNAP-5114 (red). Each point represents the SLE intensity in a single slice. Asterisks denote significant differences between control conditions and aminoguanidine + deprenyl application. Box edges represent 25th and 75th percentile, open squares represent means.

**Figure 6:**
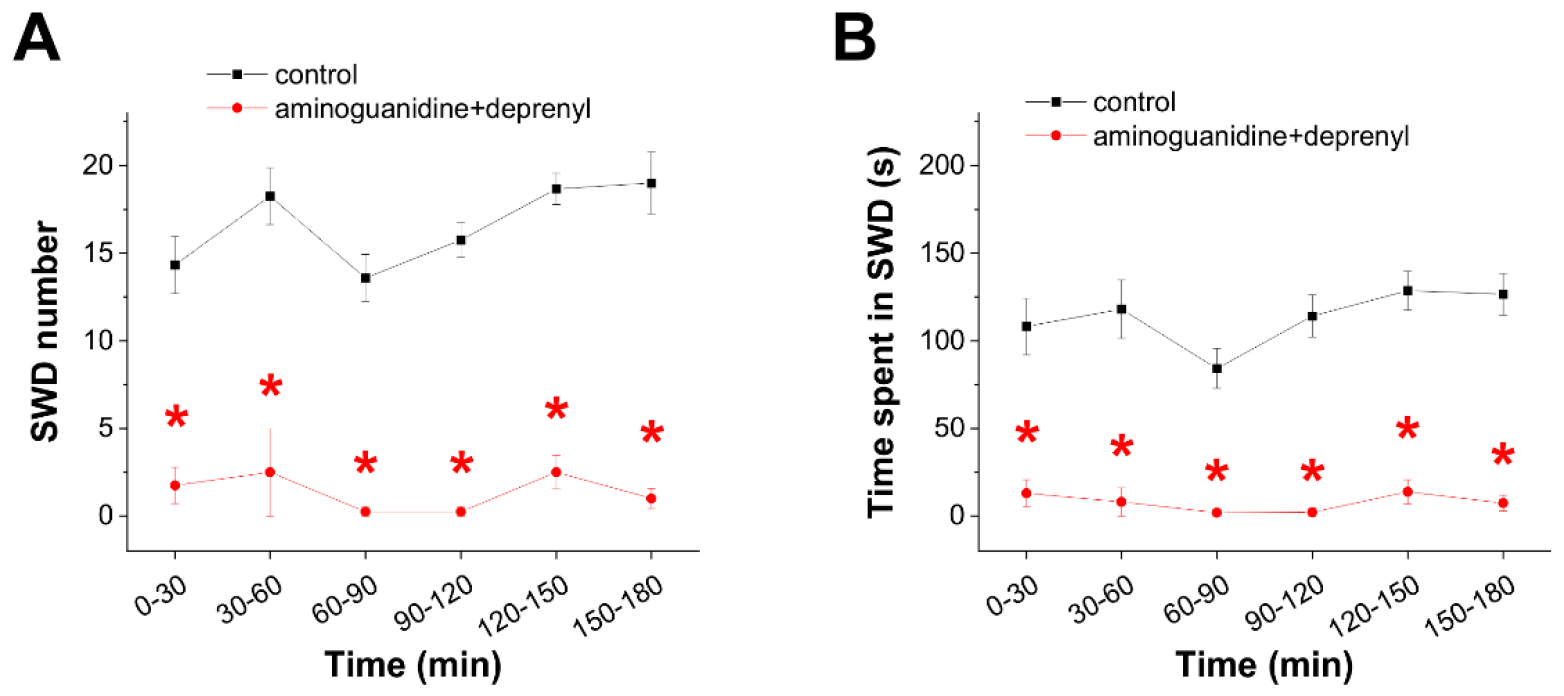

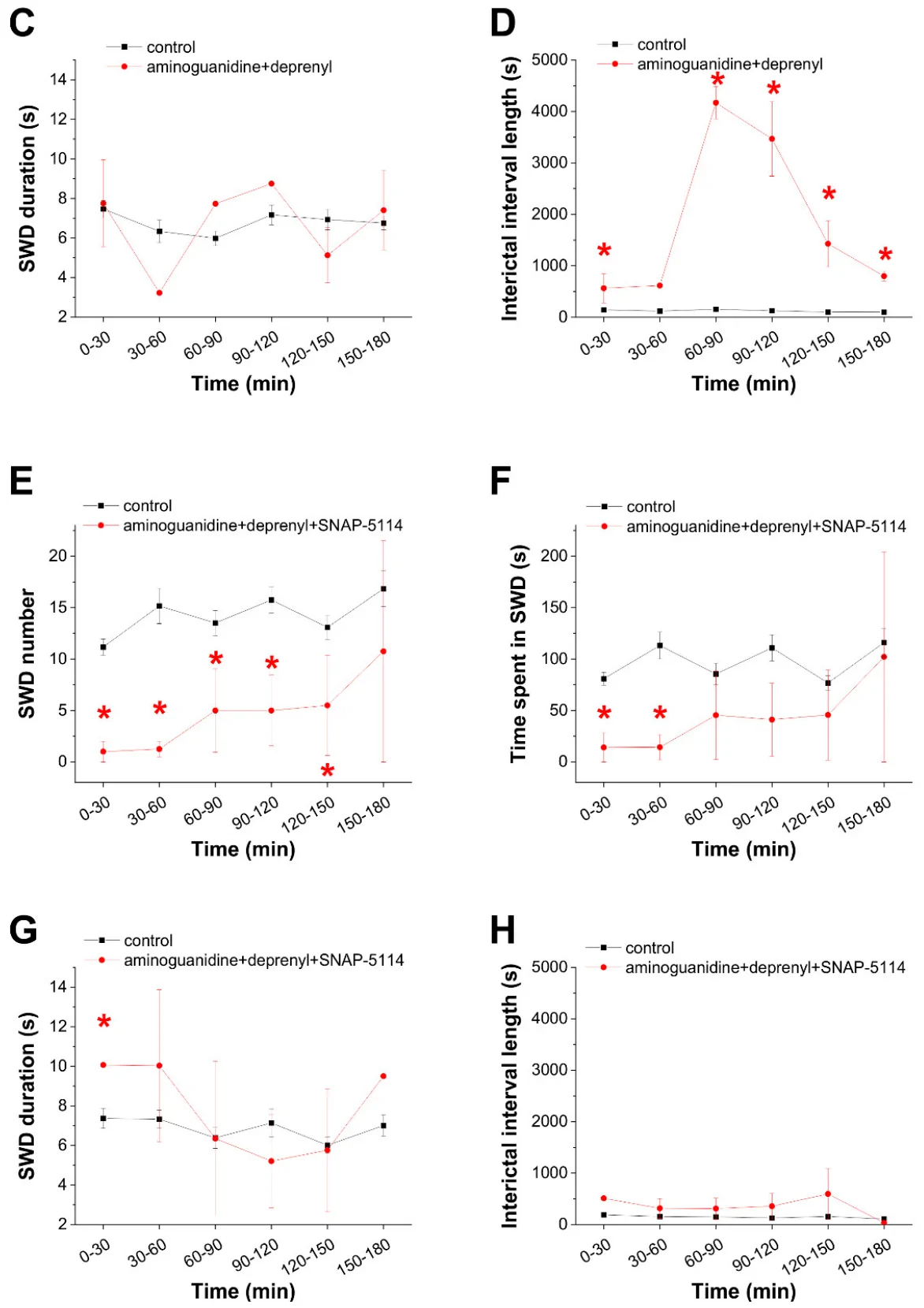
Simultaneous inhibition of monoamine oxidase B and diamine oxidase eliminates epileptiform activity and suggests the presence of an additional pathway for putrescine–GABA conversion in the in vivo WAG/Rij rat model of human absence epilepsy. Effect of simultaneous application of aminoguanidine (150 mg/kg, i.p.) + deprenyl (15 mg/kg, i.p.) on SWD number (**A**), total time spent in SWD (**B**), SWD duration (**C**) and interictal interval length (**D**). Effect of simultaneous application of aminoguanidine (150 mg/kg, i.p.) + deprenyl (15 mg/kg, i.p.) + SNAP-5114 (20 mg/kg, i.p.) on SWD number (**E**), total time spent in SWD (**F**), SWD duration (**G**) and interictal interval length (**H**). Asterisks denote significant differences between control conditions and drug applications.

Surprisingly, simultaneous MAO-B and DAO inhibition completely eliminated the appearance of SLEs in five of six slices in the low-[Mg^2+^] epilepsy model (Figure 5A,C). The average duration of SLEs significantly decreased from 93.9 ± 8.5 s measured under control conditions to 28.2 ± 7.2 s in the presence of 100 µM aminoguanidine + 10 µM deprenyl (*n* = 6 slices from 4 animals, *p* < 0.001) (Figure 5B). A similar effect was observed in the absence epilepsy model WAG/Rij rats in vivo, where both the SWD number and the total time spent in SWDs were almost completely abolished after intraperitoneal application of 150 mg/kg aminoguanidine and 15 mg/kg deprenyl (*n* = 4 animals) (Figure 6A,B). The interictal interval also markedly increased (Figure 6D).

To investigate whether this effect reflects an additional putrescine–GABA conversion pathway or a GABA-independent action of putrescine, we attempted to antagonize the antiepileptic effect of aminoguanidine plus deprenyl with 1-{2-[tris(4-methoxyphenyl)methoxy]ethyl} piperidine-3-carboxylic acid (SNAP-5114), a specific inhibitor of the astrocytic GAT-2/3 transporters. SNAP-5114 largely restored epileptiform activity both in the low-[Mg^2+^] in vitro model (100 µM) (Figure 5A,E,F) (*n* = 6 slices from 2 animals) and in WAG/Rij rats in vivo (20 mg/kg i.p.) (Figure 6E–H) (*n* = 4 animals), demonstrating that the mechanism emerging after MAO-B and DAO blockade modulates epilepsy through astrocytic GABA release. Importantly, we previously showed that 100 µM SNAP-5114 applied alone in hippocampal slices during established seizure-like activity increased SLE duration by about a third relative to control [12]. In WAG/Rij rats, 20 mg/kg SNAP-5114 given alone only slightly increased SWD appearance [10]. Because GAT-2/3 blockade [23] removes tonic inhibitory current regardless of its putrescine-dependent or -independent origin, these modest single-drug effects indicate that although GAT-2/3 blockade is itself mildly proconvulsant in both models, its effect is well short of the near-complete restoration of epileptiform activity observed here after aminoguanidine and deprenyl application. This argues against the reversal being explained solely by a putrescine-independent increase in network excitability.

### 3.5. Effect of Ornithine Decarboxylase Inhibition on Epileptic Activity

The rate-limiting step in the polyamine metabolism is the synthesis of putrescine from ornithine [24,25]. Therefore, it is plausible to hypothesize that the amount of putrescine that can be used to synthesize GABA in astrocytes can be controlled by modulating the activity of ornithine decarboxylase (ODC), the enzyme that catalyzes the ornithine–putrescine conversion. To assess this hypothesis, we evaluated whether the application of difluoromethylornithine (DFMO, a specific ODC inhibitor) [26,27] may lead to reduced putrescine concentration and therefore an increase in epileptiform activity.

**Figure 7:**
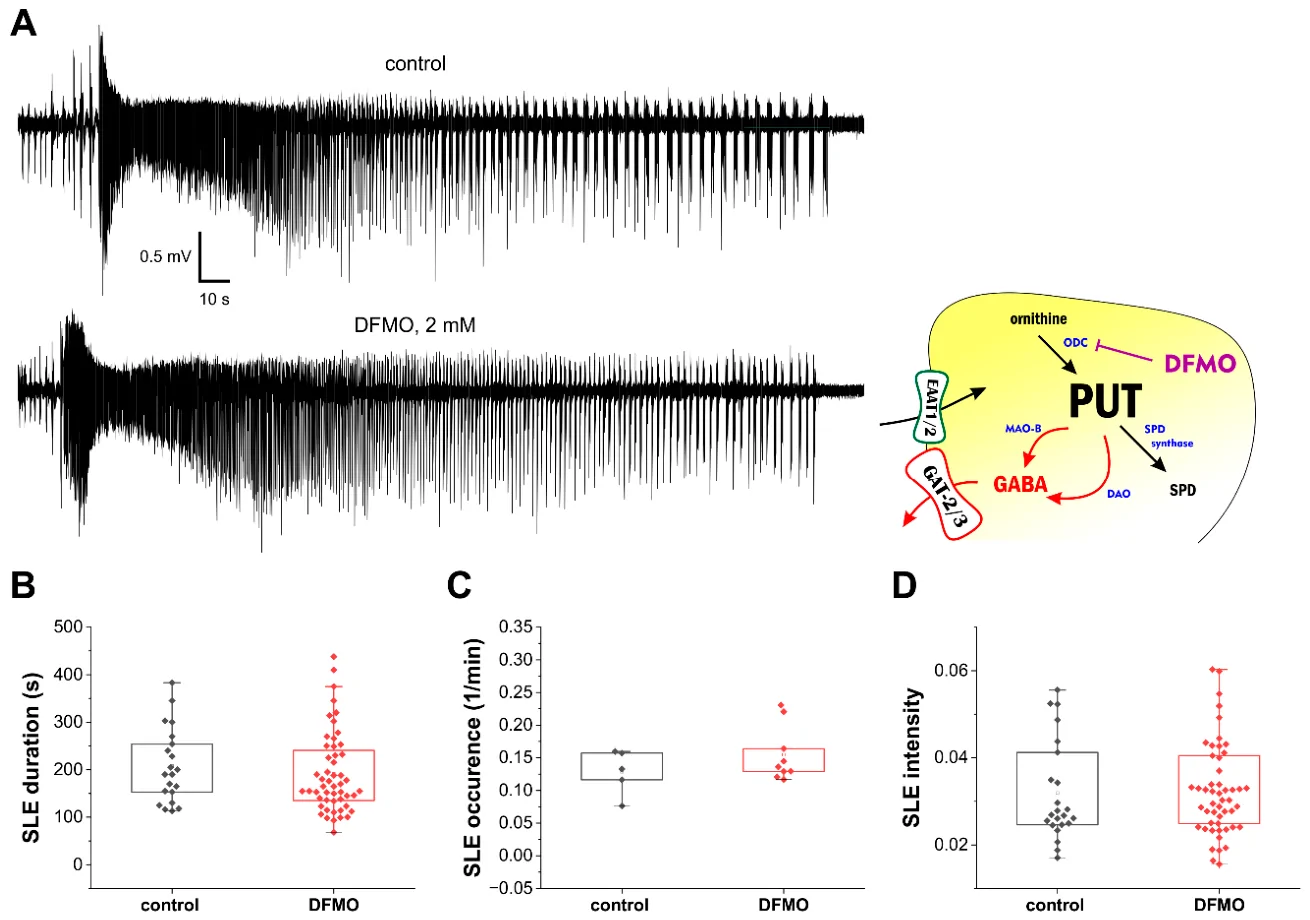
Specific inhibition of ornithine decarboxylase does not affect epileptiform activity in the low-[Mg^2+^] epilepsy model. (**A**) Representative extracellular field potential recordings of seizure-like events (SLEs) induced by low-[Mg^2+^] ACSF in the CA3 region in control slices (top) and in slices pre-treated with 2 mM difluoromethylornithine (DFMO) (bottom). (**B**) Average duration of SLEs in control slices (black) and in slices pre-treated with 2 mM DFMO (red). Each point represents the duration of a single SLE. (**C**) Number of SLEs per minute in control slices (black) and in slices pre-incubated with 2 mM DFMO (red). Each point represents the SLE occurrence in a single slice. (**D**) SLE intensity, defined as the standard deviation of the field potential in control slices (black) and in slices pre-incubated with 2 mM DFMO (red). Each point represents the SLE intensity in a single slice. Box edges represent 25th and 75th percentile, open squares represent means.

However, we did not observe any significant change in the duration (Figure 7A,B), number (Figure 7C) or intensity (Figure 7D) of SLEs (*p* = 0.45, *p* = 0.26 and *p* = 0.37, respectively) following pre-treatment of hippocampal slices with 2 mM DFMO for 2-6 hours (*n* = 9 slices from 3 animals) compared to control slices (*n* = 5 slices from 3 animals).

## 4. Discussion

Disturbances in polyamine homeostasis have long been associated with epilepsy [10,28,29,30], yet it remains unresolved as to which steps of putrescine metabolism can be effectively targeted to influence epileptiform activity. Addressing this question is particularly important given the emerging view that astrocytes can both promote and restrain epileptic activity through neurotransmitter recycling and metabolic regulation [31,32,33].

### 4.1. Putrescine as an Anticonvulsant Metabolic Hub

**Figure 8:**
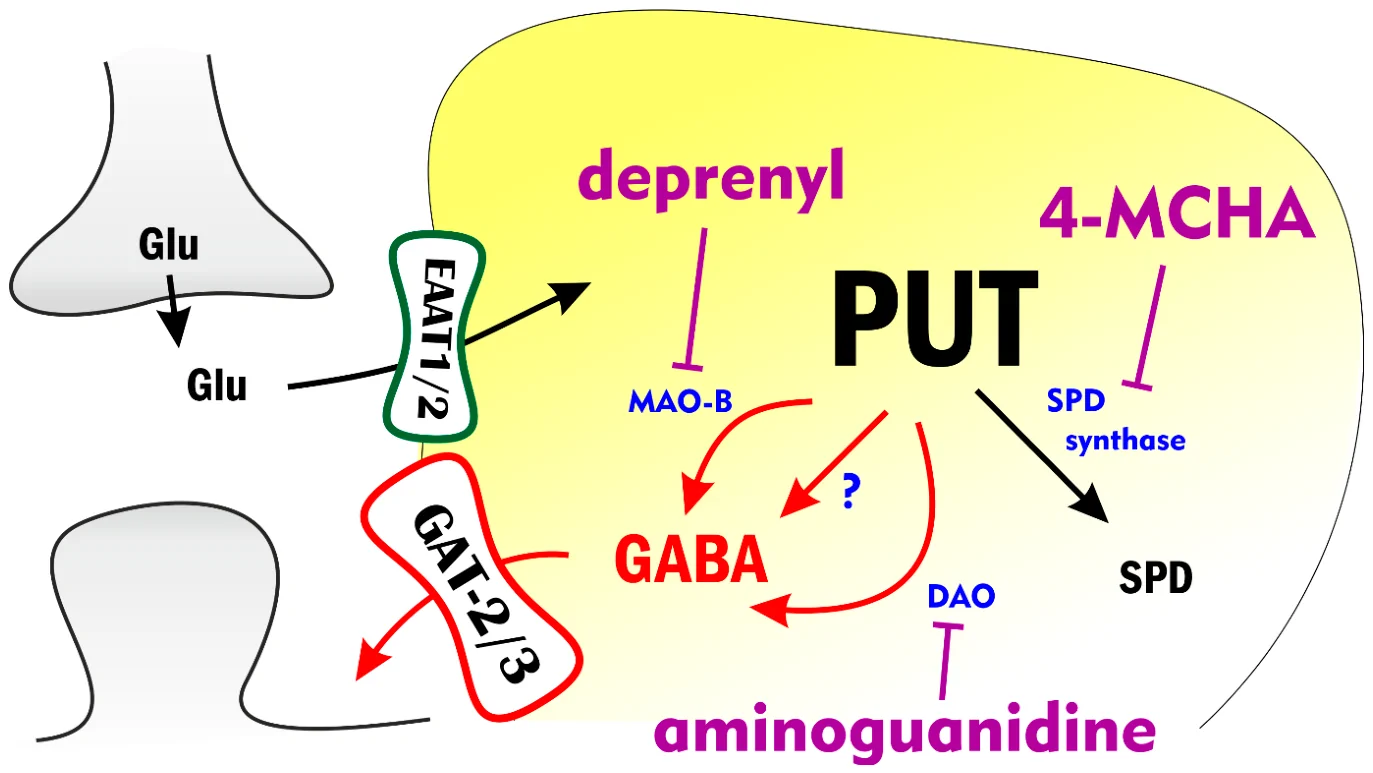
Putrescine as an anticonvulsant metabolic hub. Astrocytic polyamine metabolism affects network excitation by multiple routes. Putrescine (PUT) is converted to GABA predominantly by monoamine oxidase B (MAO-B). The astrocytic GABA is released by the reverse operation of GAT-2/3 transporters and increases tonic inhibition on neurons. PUT is also metabolized to spermidine (SPD) through SPD synthase. In addition, PUT is expected to be converted to GABA by an unknown additional mechanism, since simultaneous inhibition of MAO-B and DAO eliminates seizure appearance in both in vitro and in vivo models, and this antiepileptic effect can be antagonized by the GAT-2/3 inhibitor SNAP-5114.

Our results suggest that putrescine metabolism functions as a metabolic hub that can support anticonvulsant processes through multiple routes, with astrocytic mechanisms likely contributing to this effect (Figure 8). We have shown that increasing putrescine availability by different pharmacological interventions consistently suppresses epileptiform activity in both in vitro and in vivo models. In this context, it is especially important that putrescine occupies a central branch point in polyamine metabolism, serving both as a precursor of higher polyamines and as a substrate for GABA-producing pathways [25,34]. Importantly, attempts to disrupt individual metabolic steps in polyamine metabolism had limited or no effect. These findings indicate that anticonvulsant mechanisms arising from putrescine metabolism may be more robust to acute enzymatic perturbation than previously assumed.

The lack of significant changes following inhibition of ornithine decarboxylase with DFMO supports the notion that putrescine metabolism operates as a buffered and resilient system. This inference assumes that DFMO effectively lowered the accessible putrescine pool under our incubation conditions, although DFMO is a transport-dependent inhibitor of ODC [35,36]; therefore, it may affect the intracellular polyamine pools slowly. Short-term perturbation of individual enzymatic steps was insufficient to substantially alter the intracellular pools of putrescine or GABA that support inhibitory signaling, in line with previous observations that astrocytic putrescine concentration is maintained predominantly by uptake rather than in situ synthesis [25,34,37,38]. Consequently, disruption of a single pathway does not immediately limit the availability of substrates required for seizure-modulating mechanisms, whereas interventions that simultaneously affect multiple branches of putrescine metabolism can unmask powerful anticonvulsant effects.

One well-established route through which putrescine can exert anticonvulsant effects is its conversion to GABA in astrocytes. Astrocytic GABA can be released via the Glu-GABA exchange mechanism, in which glutamate uptake by astrocytes is coupled to GABA release through the coordinated activity of glutamate and GABA transporters [11,12]. This mechanism effectively converts network excitation into tonic neuronal inhibition and therefore provides an activity-dependent negative feedback loop during enhanced network activity. The present results further support this concept. Inhibition of spermidine synthase with 4-MCHA markedly reduced both the duration and the occurrence of SLEs in the low-[Mg^2+^] model, in accordance with our previous in vivo findings [13]. Because spermidine synthase competes with the GABA-producing pathway for putrescine, its inhibition is expected to increase the fraction of intracellular putrescine available for GABA synthesis. The resulting suppression of epileptiform activity is consistent with enhanced astrocytic GABA production and release. 

It should be noted that 4-MCHA blockade of spermidine synthase produces two simultaneous changes: an increase in putrescine availability and a depletion of spermidine. Because spermidine itself potentiates NMDA receptors at the polyamine binding site [39,40], its depletion is independently anticonvulsant and would be expected to contribute to the low-[Mg^2+^] model, in which NMDA receptor-mediated excitation drives seizure-like activity. Moreover, spermidine synthase inhibition also curtails the downstream synthesis of spermine, which is a potent modulator of gap junction conductance in astrocytes, and this action can either reduce or enhance network excitability [41,42].

Nonetheless, these findings support the idea that redirecting endogenous putrescine metabolism toward GABA synthesis can strengthen inhibitory feedback during epileptiform activity.

### 4.2. Parallel Routes of Putrescine–GABA Conversion

At the same time, our results indicate that the anticonvulsant effects of putrescine metabolism cannot be attributed to a single GABA-producing route. Inhibition of MAO-B with deprenyl produced a transient reduction in SLE duration during acute application, whereas long-term pre-treatment did not significantly alter seizure parameters. If MAO-B-dependent GABA synthesis were the sole relevant pathway, blockade of this enzyme would be expected to reduce glial GABA production and thereby facilitate, rather than attenuate, epileptiform activity. The absence of a proconvulsant effect under prolonged MAO-B inhibition, together with the lack of effect of isolated DAO blockade, therefore suggests that additional mechanisms compensate for the loss of these individual routes.

Throughout this study, compensation denotes a redistribution of metabolic flux rather than a change in the enzymes themselves. Because the MAO-B-, DAO/ALDH1A1- and PAT-SAT1/ALDH1A1-dependent routes compete for the same intracellular putrescine pool, inhibition of one branch increases substrate availability for the others without any change in enzyme abundance or catalytic activity. This is consistent with the timescale of our interventions, since transcriptional and translational remodeling of the enzymes involved is slower than the tens of minutes over which the present effects develop and are pharmacologically reversed.

Consistent with this view, simultaneous inhibition of MAO-B and DAO almost completely abolished SLEs and SWDs, and this robust anticonvulsant effect was largely reversed by the GAT-2/3 inhibitor SNAP-5114. These findings provide strong evidence that putrescine can still be converted to GABA in the absence of both MAO-B- and DAO-dependent pathways, and that the resulting GABA is released via GAT-2/3-mediated mechanisms. In this framework, the ineffectiveness of DAO inhibition alone most likely reflects compensatory engagement of MAO-B-dependent metabolism, while the strong effect of dual MAO-B/DAO blockade implies that compensation is not limited to mutual substitution between these two enzymes but also involves at least one additional putrescine–GABA conversion pathway that can be recruited under dual inhibition and contributes substantially to seizure suppression.

In the context of simultaneous MAO-B and DAO inhibition, our data raise the intriguing possibility that an additional putrescine–GABA pathway becomes preferentially engaged and exerts a strong anticonvulsant influence. Under physiological and pathological conditions, putrescine–GABA conversion in astrocytes has been primarily attributed to MAO-B-dependent degradation, with downstream oxidation steps involving ALDH1A1 and potentially sirtuin 2 (SIRT2), and a compensatory contribution of DAO during chronic MAO-B inhibition [20,37]. However, the near-complete suppression of SLEs and SWDs observed when both MAO-B and DAO are blocked, together with the ability of SNAP-5114 to largely restore epileptiform activity, suggest that alternative enzymatic routes of putrescine catabolism can still generate GABA that is released via GAT-2/3-dependent mechanisms. Although our experiments were not designed to identify the specific enzymes involved, several candidates can be considered based on previous work on astrocytic putrescine metabolism. One possibility is that additional amine or polyamine oxidases upstream of MAO-B and DAO, such as putrescine acetyltransferase (PAT/SAT1), in combination with downstream aldehyde dehydrogenases such as ALDH1A1, provide parallel routes to aminobutyraldehyde and GABA under conditions of altered redox state or enzyme inhibition. It is important to note that a direct pharmacological test of PAT/SAT1 function is currently hindered by the lack of sufficiently selective inhibitors, because available compounds show substantial off-target effects, including inhibition of GABA transaminase [43]. Another hypothesis is that increased putrescine availability during combined MAO-B/DAO blockade enhances flux through noncanonical polyamine pathways that converge on GABA production via incompletely characterized oxidases or dehydrogenases, potentially including yet-unidentified mitochondrial or peroxisomal enzymes acting on putrescine-derived intermediates.

The mechanism by which putrescine-derived GABA reaches the extracellular space also allows an alternative reading of these experiments. Because the Glu-GABA exchange is driven by glutamate uptake, a change in glutamate transport could in itself alter astrocytic GABA release, and part of the GABA that leaves the astrocyte during inhibition of MAO-B and DAO may come from the pre-existing intracellular pool rather than from GABA newly synthesized from putrescine. The present experiments were not designed to separate these two sources, and the two are not mutually exclusive, since for whichever fraction is released acutely, its replenishment depends on continued putrescine metabolism. Two of our observations are relevant here: SNAP-5114 restored epileptiform activity when it was applied after the suppression had already been established, and in vivo the suppression was maintained throughout the 30–210 min evaluation period that followed a single injection. The effects of 4-MCHA and of exogenous putrescine, which increase putrescine availability without inhibiting any oxidase, also indicate that an increased putrescine supply contributes in its own right. Separating the two sources directly would require measurements that distinguish preformed vs. newly synthesized astrocytic GABA, because blocking glutamate uptake removes the trigger of release in either case and is itself proconvulsant.

### 4.3. Limitations and Future Directions

Two limits of this interpretation should be stated. The first concerns the level at which compensation acts. Our experiments were designed to detect the redistribution of putrescine between competing catabolic routes, not changes in the amount of the enzymes that catalyze them. Whether sustained interference with putrescine catabolism also alters the expression of these enzymes was not examined here, although it is unlikely to have contributed to the present observations: drugs were bath-applied during continuous recording, the longest in vitro exposure was a 2–6 h pre-incubation, and in vivo, a single intraperitoneal dose was given, with SWDs evaluated between 30 and 210 min.

The second limit concerns glutamate decarboxylase (GAD). Unlike enzyme abundance, GAD activity can change within minutes to hours through apo-/holo-enzyme cycling, cofactor availability and phosphorylation [44], so a compensatory increase in GAD-dependent GABA synthesis cannot be excluded on kinetic grounds. Neither deprenyl nor aminoguanidine is known to act on these regulatory steps or on the supply of glutamate, and the reversal of the anticonvulsant effect by SNAP-5114 indicates release through astrocytic GAT-2/3 rather than from the vesicular pool that enhanced neuronal GAD activity would primarily supply. Astrocytic GABA, characterized so far, has also been found to be largely GAD-independent. A contribution of astrocytic GAD nevertheless remains possible, and it is not easily tested: GAD transcript, protein and homogenate activity are dominated by the neuronal compartment, and GAD inhibitors such as 3-mercaptopropionic acid or allylglycine are themselves proconvulsant [45].

Future experiments combining metabolic tracing of putrescine-derived carbon with selective inhibition or genetic manipulation of candidate enzymes such as PAT/SAT1, ALDH1A1 and SIRT2 in astrocytes will be required to determine whether the robust anticonvulsant effect observed under dual MAO-B/DAO blockade reflects recruitment of a specific alternative putrescine–GABA pathway or a broader shift in polyamine metabolism that favors tonic inhibition.

The steps of this pathway differ considerably in how accessible they are pharmacologically. The two catabolic branches are the best served. MAO-B is inhibited by selegiline, rasagiline and safinamide, all in clinical use in Parkinson’s disease, and by the selective reversible inhibitor KDS2010, which was developed to avoid the loss of astrocytic GABA that has reached phase 2 trials in Alzheimer’s disease. DAO is inhibited by aminoguanidine, taken as far as phase 3 in diabetic nephropathy before development was discontinued. Shifting putrescine towards GABA synthesis by inhibiting spermidine synthase is, in contrast, an entirely preclinical option: 4-MCHA is the best-characterized compound and alters tissue polyamine levels in rodents. Tool compounds do exist for the steps downstream of putrescine: MDL 72527 for the polyamine oxidases, AK-7 for SIRT2 [46] and NCT-501 for ALDH1A1 [47], so these branches can be dissected pharmacologically even where, as for PAT/SAT1, no selective inhibitor is available.

Raising the putrescine pool itself is less straightforward. Putrescine crosses the blood–brain barrier poorly, so systemic administration is not a practical route. Agmatine is a more promising precursor, since it is taken up into the brain and converted to putrescine by agmatinase. It is anticonvulsant in rodent seizure models [48], and it has been given orally to patients in studies of neuropathic pain.

The wider interest of these findings lies in the organization of the pathway itself. Putrescine stands at a branch point from which several routes lead to GABA, so inhibitory tone can be raised without acting on GABA receptors or on neuronal GABA synthesis. Because astrocytic GABA release through the Glu-GABA exchange is driven by the excitation it counteracts, this route works as an activity-dependent brake that engages when the network is most active, rather than as a constant dampening of excitability. With MAO-B inhibitors already in clinical use and several further steps of putrescine metabolism pharmacologically accessible, astrocytic putrescine metabolism deserves attention as a source of inhibitory tone in epilepsies that respond poorly to drugs acting on neuronal GABAergic transmission.

## 5. Conclusions

Pharmacological manipulation of putrescine metabolism suppressed epileptiform activity in two different models: the low-[Mg^2+^] model of focal epileptiform activity in vitro and the spike–wave discharges of WAG/Rij rats in vivo. Increasing the availability of putrescine by inhibiting spermidine synthase reduced both the duration and the appearance of seizure-like events, and simultaneous inhibition of MAO-B and DAO almost completely abolished seizure-like events in vitro and spike–wave discharges in vivo. The latter effect was largely reversed by blockade of astrocytic GAT-2/3 transporters, showing that putrescine is still converted to GABA when both oxidative routes are inhibited and that the GABA formed reaches the extracellular space through astrocytic GABA transporters.

We conclude that putrescine acts as a metabolic hub supplying GABA through several parallel routes that can substitute for one another so that the loss of any single route is compensated while their combined engagement carries substantial anticonvulsant potential. Raising putrescine availability, therefore, appears to be a promising method in future drug development for epilepsies that respond poorly to drugs acting on neuronal GABAergic transmission.

## Data Availability

The data that support the findings and the MATLAB (R2024a) script used to identify spike–wave discharges on in vivo recordings are available at https://figshare.com/s/e1822b5bb60d11419acf (accessed on 6 September 2026).

## Abbreviations

4-MCHA	trans-4-methylcyclohexylamine
AED	anti-epileptic drug
ALDH1A1	aldehyde dehydrogenase 1 family member A1
DAO	diamine oxidase
DFMO	difluoromethylornithine
GABA	γ-aminobutyric acid
Glu	glutamate
MAO-B	monoamine oxidase B
ODC	ornithine decarboxylase
PUT	putrescine
SLE	seizure-like event
SNAP-5114	1-{2-[tris(4-methoxyphenyl)methoxy]ethyl}piperidine-3-carboxylic acid
SWD	spike–wave discharge
